# Redes sociales como escenarios para la visibilización de las violencias basadas en género durante la pandemia de covid-19 en Colombia

**DOI:** 10.18294/sc.2024.4601

**Published:** 2024-04-02

**Authors:** Doris Elena Muñoz Zapata, Johanna Marcela Osorio Franco

**Affiliations:** 1 Comunicadora Social, Periodista. Magíster en Comunicación y Educación. Doctora en Ciencias Sociales. Docente, Facultad de Comunicación Social-Periodismo,Universidad Pontificia Bolivariana, Medellín, Colombia. doris.munoz@upb.edu.co Universidad Pontificia Bolivariana Facultad de Comunicación Social-Periodismo Universidad Pontificia Bolivariana Medellín Colombia doris.munoz@upb.edu.co; 2 Comunicadora Social, Periodista. Licenciada en Matemáticas. Diseñadora Instruccional y de Experiencias de Aprendizaje, Institución Universitaria Digital de Antioquia, Medellín, Colombia. johanna.osoriof@gmail.com Institución Universitaria Digital de Antioquia Institución Universitaria Digital de Antioquia Medellín Colombia johanna.osoriof@gmail.com

**Keywords:** Violencia de Género, Pandemia de COVID-19, Redes Sociales, Colombia, Gender-Based Violence, COVID-19 Pandemics, Social Networks, Colombia

## Abstract

El propósito de esta investigación es identificar las problematizaciones predominantes en cuentas feministas colombianas de Instagram, sobre las violencias basadas en género durante el primer año de la pandemia de covid-19. Desde un enfoque cualitativo, se realizó una etnografía digital basada en los preceptos de la netnografía, como alternativa para detallar los mundos sociales construidos a partir de los grupos en línea. A partir de las observaciones realizadas en el marco del trabajo de campo en línea se seleccionaron 50 contenidos de @lainsumisa y 20 contenidos de @feministasenconstrucción, publicados entre marzo de 2020 y marzo de 2021, los cuales se analizaron mediante técnicas de análisis del discurso. Entre los hallazgos se discuten las siguientes categorías emergentes: el trabajo no remunerado en el hogar, el acoso romantizado, la gordofobia y la vulneración a las mujeres negras y racializadas. Al respecto, se plantea el ciberactivismo como una oportunidad para el surgimiento de colectivos y redes de apoyo para las mujeres que luchan por la equidad de género y por sus derechos, hacia el cuestionamiento de ideas patriarcales que atentan contra su bienestar.

## Abstract (en)

The purpose of this research is to identify predominant problematizations in Colombian feminist Instagram accounts regarding gender-based violence during the first year of the COVID-19 pandemic. Employing a qualitative approach, a digital ethnography based on the principles of netnography was conducted as an alternative to detail the social worlds constructed from online groups. Fifty pieces of content from @lainsumisa and twenty pieces of content from @feministasenconstrucción, published between March 2020 and March 2021, were selected based on observations made within the online fieldwork framework. These were analyzed using discourse analysis techniques. The findings discuss the following emerging categories: unpaid household work, romanticized harassment, fatphobia, and the violation of Black and racialized women’s rights. In this context, cyberactivism is presented as an opportunity for the emergence of collectives and support networks for women advocating for gender equality and their rights, towards questioning patriarchal ideas that jeopardize their well-being.

## INTRODUCCIÓN

A partir de la emergencia sanitaria ocasionada por el covid-19, las violencias basadas en género se agravaron en distintos países del mundo^1^, ya que la pandemia y ciertas medidas gubernamentales como el confinamiento profundizaron las desigualdades de género, además, de las propias ocasionadas por sistemas patriarcales y neoliberales que no han sido confrontados. En Colombia, durante 2020, la Defensoría del Pueblo atendió 1.617 casos de violencia basada en género en distintos departamentos del país^2^ y ocurrieron 630 feminicidios, 56 más con respecto al 2019, año en el que se reportaron 574 feminicidios^3^, lo que indica que la situación de la violencia contra las mujeres empeoró. De hecho, las cifras se incrementaron en el 2020, año en el que se registraron 44.614 casos de violencia basada en género (feminicidios, violencia sexual y violencia de pareja) y, en el 2021, el aumento fue del 19%, con 55.582 casos^4^. 

Asimismo, el informe del año 2020, sobre la situación de las mujeres y la población con orientaciones sexuales e identidades de género diversas (OSIGD), refugiada y migrante en Colombia^2^ arrojó que los tipos de violencia física (18%), sexual (6%), psicológica (42%), patrimonial (6%) y económica (27%) fueron los que más se presentaron al interior de los hogares. Este informe^2^ también explicó que se presentaron casos en los que, por cuenta de los precarios ingresos económicos, algunas mujeres estuvieron expuestas a la escasez alimentaria, la habitabilidad de calle, la violencia sexual, la afectación a servicios de salud y de métodos de planificación. Además, en esta época, las mujeres se vieron obligadas a quedarse en casa y corrieron un mayor riesgo de sufrir violencia, el confinamiento las obligó a refugiarse con sus agresores, desprotegiéndolas, pues

…si bien las medidas de bloqueo ayudan a limitar la propagación del virus, las mujeres y las niñas que sufren violencia en el hogar se encuentran cada vez más aisladas de las personas y los recursos que pueden ayudarlas.^5^

Y, en efecto, en 2020, según la Comisión Económica para América Latina y el Caribe (CEPAL) “se registró una contundente salida de mujeres de la fuerza laboral, quienes, por tener que atender las demandas de cuidados en sus hogares, no retomaron la búsqueda de empleo”^6^. Sin contar que las mujeres tuvieron una carga más elevada, pues se duplicaron sus tareas en el hogar más sus ocupaciones o labores externas, tal como lo destaca el periódico local El Colombiano^7^ sobre las mujeres de la ciudad de Medellín, al afirmar que fueron ellas quienes vivieron los más altos impactos económicos y sociales en su calidad de vida. 

En este panorama, es claro que si bien existen estudios que evidencian que las redes sociales se han convertido en plataformas que fomentan, propagan y difunden el ciberacoso y las violencias machistas^8^, no se puede desconocer su rol de apertura y cabida a nuevas reflexiones, posicionándose como nuevos escenarios, en lo que respecta a los temas de género, que permiten construir otro tipo de discurso el cual, en gran medida, busca crear conciencia frente a esas problemáticas y sus orígenes^9^^,^^10^^,^^11^^,^^12^^,^^13^.

Las mujeres están usando sus conocimientos y experiencias para crear lugares nuevos y distintos […] Los movimientos emergentes de mujeres desde la web, como Políticas de Lugar, develan su poder y su lucha política en estos cuatro escenarios de poder, y […] redefinen su incidencia en lo público y lo privado a través de las redes sociales.^14^

### Antecedentes del ciberactivismo

El ciberactivismo surge en la búsqueda de consolidación de movimientos sociales en las plataformas digitales para responder a problemáticas específicas y situadas, y desencadenar distintos modos de activismo desde las redes sociales, como espacios de difusión, creación de contenidos y acompañamiento; de esta manera, el ciberactivismo se ha valido del auge de estas redes y ha “alimentado el surgimiento de colectivos virtuales que, algunas veces, son parte de un continuo de los grupos sociales *offline* y, algunas otras, aparecen como grupos “endémicos” y exclusivos de los espacios digitales”^15^. 

Entre los activismos promovidos en dichos escenarios, se encuentran los centrados en asuntos de género y sustentados en los feminismos, entendidos como:

…teoría política y un movimiento social que busca la igualdad de derechos entre hombres y mujeres. Es un movimiento social porque lo forman muchas mujeres y personas organizadas para cambiar la sociedad. Es una teoría política porque hace una propuesta de cómo debe ser una sociedad más justa y más respetuosa. En todos los momentos históricos y en todos los lugares del mundo, hay mujeres que se organizan para enfrentarse al poder.^16^

En la convergencia entre los activismos posibles en escenarios digitales y las búsquedas feministas surge el ciberfeminismo que es “una ventana abierta para acabar con el sistema patriarcal, lo que significa procurar nuevos escenarios para conseguir la igualdad de derechos entre hombres y mujeres”^17^. Básicamente, “el ciberfeminismo es utilizar Internet y las nuevas tecnologías para la lucha feminista”^16^.

Dentro de las problemáticas que encaran los feminismos están aquellas generadas por las violencias basadas en género, las cuales se agudizaron y potenciaron durante la pandemia^18^, tal como lo muestran los siguientes datos:

…63% de las mujeres encuestadas dijeron que ellas mismas u otras mujeres que conocen habían experimentado alguna forma de violencia contra las mujeres. 11% dijeron que esto ha empeorado como resultado de la pandemia. 21% de las mujeres se sienten inseguras en su hogar. 20% dijeron que el conflicto entre adultos se ha vuelto más frecuente como resultado de la pandemia […] 43% de las mujeres piensan que la experiencia de abuso verbal o físico a manos de su pareja es común para las mujeres de su comunidad. 43% dijeron que esto ha empeorado a causa de la pandemia.^19^

En el caso concreto del departamento de Antioquia, se devela que:

En el informe XIX sobre la violación de los derechos humanos de la mujer en Antioquia, realizado por la*Corporación Vamos Mujer y la Corporación para La Vida, Mujeres que Crean*, se evidencia que el Estado no logró prevenir ni atender el aumento de vulneración de derechos. 4886 casos de violencia de pareja en Antioquia, y en Medellín 2263 casos de delitos sexuales en el último año.^20^

Además, de acuerdo con el informe elaborado por la Defensoría del Pueblo Colombia^2^ sobre la situación de las mujeres y la población OSIGD, refugiada y migrante en Colombia del 2020, los tipos de violencia más frecuentes en los hogares fueron la psicológica, la física y la económica. De hecho,

…la violencia psicológica constituye el mayor porcentaje dentro de los tipos de violencia expuestos, y es transversal a todos estos, ya que, cualquier hecho que cause daño a la persona en cualquiera de sus formas, desencadena también un daño y sufrimiento psicológico. Este tipo de violencia hace referencia a comentarios intimidatorios, acoso, amenazas, aislamiento, desprecio y humillación, entre otras.^2^

Comprender la violencia machista es ir más allá de estar enterado de las cifras de violencia, sentirse indignado cuando ocurren eventos asociados o identificar cuándo una mujer es violentada. En la problematización de dicha violencia, el mensaje es una apuesta por la creación de conciencia social, respeto y prudencia en comentarios, señalamientos o juicios de valor hacia las víctimas; pero, de manera central, es una apuesta por la empatía con las víctimas, con el propósito de evitar a toda costa revictimizarlas o violentarlas de otras maneras.

La revictimización o victimización secundaria se da cuando la misma víctima, aparte del ocasionado por el delito, sufre daño posterior causado por los impartidores de justicia, por la policía, jueces, voluntarios y trabajadores del sistema penal, y por la misma sociedad, incluyendo familiares, comunidades o medios de comunicación.^21^

Es evidente que la violencia como forma masculina y patriarcal de relacionarse con el otro ha creado una proyección en la construcción del amor romántico y, por lo tanto, este se ha asociado con poseer a la otra persona, dominarla y ser dueña de ella, deshumanizándola desde la violencia física, sexual, psicológica, entre otras. Para ejemplificar esto, se tienen miles de casos de feminicidios en todo el mundo. Solo en Colombia, en el 2021, se registraron 622 feminicidios, situaciones en las que la mujer se asumió como bien masculino, sin autonomía y deshumanizada^22^. 

Considerando este referente conceptual, el objetivo principal de esta investigación es identificar las problematizaciones predominantes en cuentas feministas colombianas de Instagram, sobre las violencias basadas en género durante el primer año de la pandemia de covid-19.

## METODOLOGÍA

Esta investigación cualitativa hace uso de la etnografía digital^23^, para realizar un trabajo de campo *en línea*, asumir los espacios digitales como escenarios de socialización y llevar a cabo análisis del discurso en los contenidos publicados en una red social, desde los preceptos de la netnografía^24^, como alternativa para detallar los mundos sociales construidos a partir de los grupos en línea. 

Desde esta perspectiva, se realizaron observaciones entre el 12 de febrero de 2022 y el 15 de marzo de 2022 en las cuentas de Instagram @lainsumisa y @feministasenconstrucción, en las que fueron consideradas las publicaciones realizadas durante el primer año de la pandemia de covid-19 en Colombia, es decir, entre marzo del 2020 y marzo del 2021. Se tomaron apuntes de elementos a destacar, los cuales facilitaron al análisis.

Las dos cuentas de Instagram seleccionadas cuentan con perfiles públicos. La creadora de contenidos de la cuenta @lainsumisa es Paola Tatiana Duque Rueda (periodista y estudiante -al momento de la investigación- de la especialización en Violencia(s) de género: Estado, políticas públicas y movimientos sociales). Promueve la idea de comunicación libre de violencia simbólica, maternidad feminista y contenidos de la cultura masiva desde un enfoque de género y ofrece capacitaciones y talleres con temas asociados. 

Por su parte, la cuenta @feministasenconstrucción construye contenidos sobre el feminismo, con el propósito de aportar y ayudar a otras mujeres a salir del closet feminista. Inicialmente, nace como blog, impulsado por un equipo de mujeres conformado por Olga Bohórquez, Mema Carrillo, Andrea Dávila, Daniela Ayala, Mariana Garcés, Laura Camila López, Sara Gaviria y Melissa De La Hoz. Además, cuenta con el apoyo de la Fundación Cideem, que trabaja por la equidad de la mujer.

Para esta investigación, se tomaron como muestra 50 contenidos publicados en la cuenta @lainsumisa y 20 contenidos publicados en la cuenta @feministasencontrucción, y las interacciones de las personas usuarias de cada cuenta. Los parámetros para la selección de los contenidos fueron que estuvieran en el margen temporal delimitado en esta investigación y que se relacionaran con violencias basadas en género en el marco de la pandemia de covid-19. 

Antes de comenzar la investigación, se contactó a las creadoras de contenido Paola Tatiana Duque Rueda (de @lainsumisa) y Laura Camila López (de @feministasenconstrucción) y se les informó sobre el presente trabajo de investigación, su objetivo y alcance, y se obtuvo el consentimiento para realizarla. 

El cuerpo de contenidos seleccionados se analizó mediante técnicas de análisis del discurso^23^, que permiten aproximarse a una realidad social, en tanto las personas -como sujetos de un discurso particular, ubicado en un período histórico- generan sus propios sentidos, de manera tal “que los discursos mismos construyen las posiciones-sujeto desde las cuales ellos se vuelven significativos y tienen efectos”^25^.

El análisis del discurso se realizó mediante matrices analíticas y gráficos para la comprensión de las violencias basadas en género durante el primer año de la pandemia de covid-19 en Colombia. El análisis se apoyó en el programa NVivo, versión 11, que permitió visibilizar las relaciones inmersas en las categorías analíticas, así como sus componentes. La denominación de dichas categorías se planteó a partir de los conceptos desarrollados en las publicaciones que buscaban visibilizar las principales violencias basadas en género que destacaron durante la pandemia de covid-19 en Colombia. Del proceso analítico de los datos de ambas cuentas, surgieron las siguientes categorías emergentes:

- *Trabajo no remunerado en el hogar:* se propuso para hacer visible la violencia económica de la que son víctimas las mujeres.
- *Acoso romantizado:* visibiliza la violencia afectiva que experimentan las mujeres en los diferentes espacios que habitan.
- *Gordofobia*: asumida a partir de la violencia psicológica y verbal que denigra los cuerpos a partir de estereotipos físicos.
- *Visibilidad de la mujer negra y racializada*: se propuso como una manera de reivindicar, desde la interseccionalidad, a aquellas mujeres que son violentadas de diversas maneras y por diferentes condiciones.

También, se analizaron las interacciones del público con los contenidos publicados en las dos cuentas de Instagram. La interacción verbal y la comunicación pertenecen al nivel microsocial^26^, lo que permite entender que, en el mundo de las redes sociales, mediado por lo digital, las interacciones crean y establecen una microsociedad activa. Para efectos de esta investigación se asumieron dos niveles de interacción:

- *Primer nivel de interacción*: en este nivel se tuvieron en cuenta el número de visualizaciones y de *Me gusta* en las publicaciones.
- *Segundo nivel de interacción*: en este nivel se revisaron los calificativos, es decir, las palabras clave que califican las publicaciones.

Al concluir el ejercicio analítico, se realizó una contrastación^27^ de los resultados con las autoras de las cuentas. En esa revisión, ambas manifestaron satisfacción por la elección de sus cuentas y expresaron evidenciar una posibilidad de reflexión frente al trabajo creativo que ellas realizaban en las redes sociales sobre la construcción de conciencia del rol de la mujer, las violencias machistas y los temas de género. 

Entendiendo que el concepto de violencias basadas en género (VBG) en pandemia en esta investigación se asume a partir de lo propuesto en el informe de la Defensoría del Pueblo del 2020 y la Asociación Palco sobre la violencia contra las mujeres durante la pandemia. Y se operacionaliza acuñada en el sexismo, el cual se asocia directamente con el machismo, la cultura patriarcal, la misoginia y la homofobia, que se fundamenta en la premisa base de que lo masculino es superior, mejor, más capaz, más útil y privilegiado en comparación con lo diferente o lo femenino^28^.

Para determinar las características generales de las personas que siguen cada cuenta, se recolectó información, de manera aleatoria, sobre los perfiles que siguen las dos cuentas, en total se registraron 10 perfiles públicos, con una edad promedio de 30 años y, en su mayoría, mujeres colombianas. Los intereses y preferencias de la mayoría se asocian con feminismo, movimientos sociales y políticos, naturaleza, mascotas, fiestas, viajes, cultura. En general, se devela que son personas con un nivel de educación media y superior, la mayoría son profesionales de carreras afines a las ciencias sociales y humanas, también se destacan modelos, asesores de estilo de vida y emprendedores.

## RESULTADOS Y DISCUSIÓN

Las cuentas de la red social Instagram @lainsumisa y @feministasenconstrucción, analizadas en esta investigación, problematizan la violencia machista durante el primer año de pandemia de covid-19 en Colombia a través de comunicaciones gráficas (piezas gráficas con texto, ilustraciones o fotografías en un carrusel de imágenes) e infografías (una sola pieza gráfica) publicadas en sus perfiles. 

La frecuencia de publicación durante el primer año de la pandemia de covid-19 fue alta en ambas cuentas. La @lainsumisa publicó, en promedio, cada tres días entre marzo de 2020 y marzo de 2021 y la cuenta @feministasenconstrucción publicó, en promedio, cada cuatro días, aproximadamente. Esto les permitió visibilizarse, atraer nuevos seguidores, brindar contenido de manera continua y posicionarse como cuentas activas.

Los *copys* que acompañan las publicaciones, que en la matriz se etiquetaron como títulos, tienen textos que exponen la postura de las creadoras de contenidos de cada cuenta frente a las distintas temáticas de cada *post*, por lo general, tales posturas están dirigidas a las mujeres y suelen ser reflexiones, críticas, cuestionamientos o llamados a la acción. Además, casi siempre están escritos en primera persona plural y se destaca el uso de emojis, *hashtags* de temas o tendencias asociadas, menciones o etiquetas de otras cuentas o personas autoras de las ilustraciones o mensajes, *call to action* o invitaciones permanentes al público para redirigirse al blog de cada cuenta, escuchar episodios de podcasts propios o recomendados, entrevistas o videos asociados a la temática.

De las publicaciones seleccionadas, en la cuenta @lainsumisa, el número más bajo en las interacciones de primer nivel (visualizaciones y Me gusta) es de 77 y el número más alto es de 5.791. Por su parte, en la cuenta @feministasenconstrucción, el número más bajo en las interacciones de primer nivel (visualizaciones y Me gusta) es de 99 y el número más alto es de 4.473.

En cuanto al segundo nivel de interacción, es decir, los calificativos otorgados por los seguidores en los comentarios de las publicaciones son muy similares en ambas cuentas, pues casi todos son elogios y palabras halagadoras. Los comentarios más frecuentes son mensajes de gratitud y reacciones con emoticones como aplausos, globos, corazón, fuego, sol, flores, puño empoderado, etc. Además, destacan palabras como: “válida”, “necesario”, “reivindicante”, “visibiliza”, “fantástico”, “sencillo”, “cuestionador”, “excelente”, “revolución”, “hermoso”, “revelador”, “brutal”, “oportuno”, “inspirador”, “me encanta”, entre otros. Mientras que, en los comentarios negativos o en desacuerdo, destacan términos como: “discutible”, “mucho texto”, “dejen de chillar”, “dejen la desigualdad con los hombres”; pero, en general, prevalecen los comentarios a favor. 

A continuación, presentamos el análisis y la discusión de las siguientes categorías emergentes: trabajo no remunerado en el hogar, acoso romantizado, gordofobia y visibilidad de la mujer negra y racializada.

### Trabajo no remunerado en el hogar

De acuerdo con datos aportados por @feministasenconstrucción, uno de los roles de género más destacado es la mujer madre y cuidadora. Al respecto, dicha cuenta resaltó el informe *Cuidado no remunerado en Colombia: brechas de género, la economía del cuidado*, el cual devela que “la producción del trabajo doméstico y de cuidados no remunerados equivale al 20% del PIB colombiano”^29^. Esto significa que las mujeres aportan más que cualquier otro sector a la economía del país, si se considera que el 78% de las horas anuales destinadas a las tareas del cuidado dentro del hogar son realizadas por las mujeres. El mensaje es contundente, la sociedad no reconoce, valora, ni remunera los cuidados domésticos, a pesar de ser un pilar fundamental de la economía.

Este panorama representa una distribución desproporcionada que aumenta la carga física y mental de las mujeres y les genera consecuencias negativas en su salud. Y, aunque hay una circular del Ministerio de Trabajo^30^ que regula el trabajo en casa, durante el aislamiento obligatorio, la carga de los hombres fue mucho menor con respecto a la de las mujeres. Ante este contexto, que fue transversal durante las publicaciones en el primer año de pandemia de covid-19 en ambas cuentas de Instagram, los roles de género y la violencia de género asociados en esta categoría no solo se problematizaron a través de la denuncia y la exposición de cifras o estudios, sino también mediante la exhortación permanente a las mujeres, y a la sociedad en general, a cuestionar, reflexionar, pensar y deconstruir prácticas y discursos de un sistema machista, utilitario, opresivo, antimaternal y niñefóbico. 

Las creadoras de contenidos de @feministasenconstrucción explican que las labores de cuidado históricamente han tenido género: el femenino.

*La crisis que atravesamos actualmente como sociedad ha exacerbado la histórica desigualdad en la división de las labores de cuidado, las cuales han estado supeditadas en mayor proporción a las mujeres*. (@feministasenconstrucción, 31 de julio de 2020, Instagram).

Las problematizaciones respecto a esta temática son relevantes y urgentes, en tanto la pandemia de covid-19 exacerbó las dificultades de las mujeres para el acceso a los recursos básicos y a la conciliación laboral, también en relación con los asuntos propios de las maternidades y la crianza^31^^,^^32^^,^^33^^,^^34^^,^^35^^,^^36^. Lo anterior afianza la feminización de los cuidados, con implicaciones en el deterioro de la calidad de vida de las mujeres en múltiples aspectos, lo que perpetúa las brechas de género que, en el caso latinoamericano, tienen amplias dimensiones^37^^,^^38^^,^^39^.

El camino que se vislumbra para esta problemática en @feministasenconstrucción es la *apuesta por la economía del cuidado*. En Colombia, de acuerdo con la Ley 1413 de 2010, la economía del cuidado hace referencia “al trabajo no remunerado que se realiza en el hogar, relacionado con mantenimiento de la vivienda, los cuidados a otras personas del hogar o la comunidad y el mantenimiento de la fuerza de trabajo remunerado. Esta categoría de trabajo es de fundamental importancia económica en una sociedad”^40^.

Para @lainsumisa, “*la economía del cuidado es la producción, cuidado y sostenimiento de la vida normalmente a cargo de las mujeres*” (@lainsumisa, 11 de abril de 2020, Instagram). La lucha por la economía del cuidado es la lucha por la economía de la vida. Una economía que han sostenido las mujeres, aquellas que han pagado el precio de la guerra, han pedido la verdad y la memoria, y defienden la paz, porque “*en tiempo de guerra, la mujer sostiene la vida*” (@feministasenconstrucción, 09 abril de 2020, Instagram).

### Acoso romantizado

El contexto de confinamiento propició que muchas mujeres pasaran más tiempo con sus agresores y estuvieran más expuestas a diferentes formas de violencias por parte de estos. En tal sentido, ambas cuentas problematizaron los límites del amor romántico con el acoso y la violencia. 

Al respecto, la cuenta @feministasenconstrucción introduce una reflexión sobre la romantización del amor de pareja:

*Hace algunos años leí que uno cambia un amor de la vida, por otro amor y otra vida, y creo que fue ahí donde comencé a entender que, la idea de un único amor de la vida, que es tu alma gemela y la única persona con la que harás click y se amarán por siempre, no es tan cierto. Entendí que, hay amores de distintas vidas; la vida que estés viviendo en ese momento. La etapa. Hay amores fantásticos, amores tóxicos, amores pacientes, amores rebeldes, hasta amores imposibles. Amor es lo que hay en el mundo, como para conformarse con tener UN solo amor de la vida. Es también por esta idea del único amor, que nos quedamos estancadas en el amor romántico y tóxico, que nos quedamos estancadas en la vida. Olvidémonos del amor de la vida. Más bien tengámosle más amor a la vida, a nosotras, a nuestros cuerpos.* (@feministasenconstrucción, 22 de mayo de 2020, Instagram)

El amor romantizado adentra un bienestar que, en muchas ocasiones, enmascara omisiones sobre actitudes o acciones violentas que deberían ser alarmantes, pero se camuflan. Algunas de ellas, son mencionadas por Laura Rodríguez, creadora de contenidos en @feministasenconstrucción:

*Si te revisa el celular, si se pone bravo cuando sales con tus amigas, si te humilla en conversaciones, sino le gusta que hables con otras personas, si toma y se pone agresivo, si te hace comentarios sobre tu cuerpo. Eso no es amor, es violencia*. (@feministasenconstrucción, 18 febrero de 2021, Instagram)

No hay certeza desde cuándo se les enseñó a las mujeres que “*se hacen rogar y los hombres deben insistir*” lo que da pie para reflexionar también sobre la romantización del acoso, dado que se ha normalizado la insistencia, el “*dime que no, pero yo sé que sí quieres*”, lo cual se convierte en una situación peligrosa y violenta.

*No es No, no importa mi ropa, no importa el lugar, no importa tu cargo de poder, no importa el tipo de relación que tengamos, no importa tu opinión sobre mi cuerpo*. (@feministasenconstrucción, 16 julio de 2020, Instagram).

Según Natalia Muñoz Ferrer, el acoso romantizado:

…es un tipo de acoso que es validado desde el amor, el romanticismo y la forma de relacionamiento entre hombres y mujeres, es un acoso que se mantiene en el tiempo y en la mayoría de los casos se da por una persona desconocida o por alguien con quien nunca se ha tenido una relación de pareja. Es un acoso que desde la mirada social y jurídica no representa ningún peligro para la mujer, pues el agresor nunca ha ejercido violencia física y cuando ha intentado acercarse es con fines románticos (tratar de darle un beso, un abrazo, tocar su mano o tomarle una foto).^41^

Este tipo de acoso es una violencia que está en el límite de dos esferas: la privada y la pública, y se relaciona con la idea de posesión y propiedad, ya que las validaciones sociales para justificar este tipo de acoso pueden analizarse desde el pacto social que sigue estando condicionado en su mayoría por lo que se conoce como el contrato sexual que reside en las relaciones de opresión y dominación que tienen los hombres sobre las mujeres y sus cuerpos: el hombre como propietario tiene el derecho de acceder a las mujeres, que terminan configurándose como objetos de deseo, a quienes pueden acceder, ellas quieran o no. Esto continúa perpetuando el binarismo de género impuesto por el patriarcado^41^. 

Lo más delicado del asunto es que aún no existe en Colombia un marco legal que penalice específicamente el acoso romantizado. Para esta “violencia invisibilizada, poco documentada y escasamente penalizada, no existe un delito a nivel penal que lo reconozca, pues como lo hemos dicho anteriormente, está naturalizado desde las relaciones entre hombres y mujeres y los roles de género establecidos socialmente”^41^, a pesar de que muchas personas cercanas a las víctimas de este tipo de acoso lo validan, minimizan y muestran como algo jocoso o sin importancia^41^. En definitiva, el acoso y la insistencia nunca son románticos. 

El camino que se dilucida para esta problemática es entender y respetar el valor del consentimiento y el deseo y la suficiencia del No. Hay actos opresivos y violentos que se han romantizado porque se ha asociado el sufrimiento con amor y, en ello, han tenido que ver mucho los ideales, roles y estereotipos difundidos por la televisión y el cine. 

Esta problematización devela la propuesta permanente a cuestionar formas, espacios y arquetipos al establecer relaciones para reinventar formas de crear lazos con otros. La apuesta debe ser “*por un amor compañero, antipatriarcal y subversivo*” (@lainsumisa, 03 mayo de 2020, Instagram).

Además, vale la pena recalcar la importancia de realmente comprender la violencia machista. Aquí, es apropiado aludir a una de las publicaciones de @lainsumisa, en la que se problematizan los roles de la mujer en una relación violenta. La típica pregunta que se les suele hacer a mujeres en estas circunstancias, ¿*por qué no te fuiste*?, es una pregunta que “*invisibiliza al hombre que violenta y al contexto que no protege a la mujer*” (@lainsumisa, 24 febrero de 2021, Instagram); pero, sobre todo, culpa a las víctimas e ignora los factores psicológicos, emocionales y materiales que le dificultaron la salida de una situación de violencia.

### Gordofobia

La gordofobia es el odio, rechazo y violencia que sufren las personas gordas por el hecho de ser gordas. Es una discriminación que está cimentada sobre prejuicios respecto a los hábitos, costumbres y salud de las personas gordas, los cuales se nutren de la creencia de que el cuerpo gordo responde a una falta de voluntad o de autocuidado, de no hacer el esfuerzo suficiente para ser delgado, motivo por el cual merece “castigo” o rechazo.^42^

De este modo, la gordofobia compone un sistema de opresión que pone al sujeto en una situación de desventaja, injusticia y exclusión, que se reproduce de forma sistemática y estructural^43^, mientras la sociedad actual está preocupada por la estética del cuerpo, la belleza y la moda, en la que se han establecido cuerpos normados, “aceptados y ofertados como símbolo de éxito, salud y disciplina, mientras que aquellos cuerpos salidos del riguroso esquema corporal hegemónico se consideran, vagos, feos y enfermos”^44^. 

Esto toma mayor relevancia si se piensa en el contexto de la red social Instagram que, como se indicó al inicio de esta investigación, le otorga un alto predominio a la imagen, prioriza estándares de belleza y valida una percepción/aspiración sobre los cuerpos al que todos deben apuntar, lo cual se refuerza con la idea de lo *ligth*, lo delgado y lo *fitness*, conceptos asociados a la disciplina, la belleza, la salud y el éxito; sobre todo, conceptos que se han usado para discriminar y etiquetar como indeseables los cuerpos diferentes, gordos, flácidos y marcados por estrías o cicatrices. En este sentido, la gordofobia es entendida como el proceso que “enuncia la expresión de odio hacia los cuerpos que no encajan en los patrones corporales normativos”^44^.

La cultura pop se ha encargado de narrar a la mujer gorda como infeliz, incompleta, con una historia limitada y poco profunda que dicta que las mujeres gordas no pueden vivir a pleno:

*El velo del sexismo y las narrativas gordodiantes han plagado la historia del cine y la televisión y es algo que hemos normalizado junto con otras opresiones estructurales*. (@lainsumisa, 29 de enero de 2021, Instagram).

Es evidente que el estereotipo de delgadez ha reinado en el cine y la televisión, promoviendo la idea de que la mujer delgada y bella es exitosa, de que el objetivo vital de las mujeres es la aceptación masculina y que la mujer gorda se acepta siempre y cuando esté al servicio del deseo masculino. Además, nos han vendido una idea de salud atravesada por el racismo y la gordofobia. La cultura popular “*ha normalizado dispositivos como las dietas. Por lo que uno de los llamados a las mujeres es a desnormalizar este estereotipo y reconocerse por fuera de la hegemonía sin culpa*” (@lainsumisa, 4 agosto de 2020, Instagram). 

Y no es para menos este llamado, durante el confinamiento ocasionado por la pandemia de covid-19, la gordofobia y el *fat shaming* (vergüenza gorda) se viralizaron en redes sociales mediante *posts* con imágenes de matoneo y burla hacia la gordura, generando problemas de autoestima y ansiedad. La viralización de este fenómeno fue tal que, según este portal, logró ser tan pandémico como el mismo coronavirus^45^. 

Este es un fenómeno que se maximiza, si se tiene en cuenta que las cifras de obesidad son alarmantes. En una nota elaborada con motivo del Día Mundial de la Obesidad^46^, según datos de la Organización Mundial de la Salud (OMS), en 2022, la obesidad afectaba a 1.029 millones de personas en el mundo, entre ellos, al menos 650 millones son adultos, 340 millones son adolescentes y 39 millones son niños. La OMS advierte, además, que para 2025 aproximadamente 167 millones de personas (adultos y niños) se volverán menos saludables debido a que tienen sobrepeso u obesidad. Estos datos, al parecer, tienen menos interés en el movimiento gordofóbico.

El camino para contrarrestar el asunto de la gordofobia es generar conciencia social y educar para “*que la gordofobia no sea preocupación, pues no es saludable, optemos porque nuestro cuerpo no sea objeto de opinión pública, lo que vale es la opinión de cada mujer sobre su cuerpo y su autoaceptación*” (@feministasenconstrucción, 07 mayo de 2020, Instagram). “Una mujer que no se gusta a sí misma no puede ser libre, y el sistema nos educa para que nunca lleguemos a gustarnos”^42^, por lo que un camino posible puede ser la autoaceptación.

### Visibilidad de la mujer negra y racializada

Frente a esta problematización, la cuenta @lainsumisa se apoya en datos de Global Health para referir cifras que dan contexto sobre la situación de las mujeres en Colombia, durante la pandemia de covid-19:

*...3 de cada 10 mujeres de 15 años en adelante no tenía ingreso propio. Por cada 100 hombres pobres, había 118 mujeres. El confinamiento incrementó la vulnerabilidad de las víctimas de violencia de género. La mitad de población femenina 53% comparado con el 74% de la masculina participa en el mercado laboral y son las mujeres con mayor presencia en baja productividad, empleos temporales, parciales, informales y autoempleo*. (@lainsumisa, 13 abril de 2020, Instagram)

Esto deja en evidencia que, durante la pandemia de covid-19, aumentó la necesidad de asistencia y apoyo a las mujeres más vulnerables, mediante enfoques de género e interseccionalidad en las políticas públicas, para internalizar los impactos sociales de la crisis que permanecen invisibles, al considerar las desigualdades simultáneas que confluyen en el caso de las mujeres negras y racializadas, en contextos latinoamericanos, así como las afectaciones directas en sus posibilidades de agencia dentro de los sistemas sociales en los que están inmersas^47^.

Adicionalmente, respecto del papel de las mujeres en el manejo del covid-19, a nivel mundial:

Es paradójico que hubo ausencia de mujeres en órganos y comités para diseñar estrategias para frenar el virus, pero conformaron la mayoría de la primera línea de la lucha contra la enfermedad, exponiéndolas en mayor proporción que a los hombres. En la composición del personal sanitario mundial, la presencia de mujeres fue del 70%. (@lainsumisa, 13 abril de 2020, Instagram)

Por lo que, además de la asistencia y el apoyo a las mujeres vulnerables, debe continuar la lucha permanente de las mujeres para ocupar un lugar, para posicionarse en roles estratégicos y de liderazgo.

Ahora bien, tanto la vulnerabilidad de las mujeres como la relegación a roles específicos y la baja participación en ciertas esferas de la sociedad (aquellas exclusivas para hombres), traen a la discusión la interseccionalidad, “*aquello que nos permite entender que a las mujeres nos atraviesan muchas otras condiciones más allá del hecho de ser mujer. Condiciones que imponen otros tipos de dominación, violencias y desigualdades*” (@feministasenconstrucción, 4 junio de 2020, Instagram). 

De esta forma, la interseccionalidad configura la forma de entender las diferentes experiencias de dominación que viven las mujeres, “la interseccionalidad parece haberse convertido en el tropo feminista más difundido para hablar ya sea de identidades o de desigualdades múltiples e interdependientes”^48^.

*Este concepto emerge en Estados Unidos, a fines de los años 80, a partir del trabajo de la abogada feminista afrodescendiente Kimberle Crenshaw, quien se encargó de visibilizar la insuficiencia de las leyes estadounidenses para abordar las múltiples dimensiones de la opresión experimentadas por las mujeres afrodescendientes.* (@feministasenconstrucción, 4 junio de 2020, Instagram).

En esta problematización, la reflexión está dirigida a las mismas mujeres, a las blancas, las heterosexuales profesionales, las citadinas y a las feministas, para que no ocurra como en los inicios del feminismo europeo blanco homogeneizador, ni se homogenice el sujeto político del feminismo; para entender que hay otras cientos de luchas de mujeres que atraviesan el feminismo, luchas que también se deben abrazar, estudiar, respetar y asegurar que sean escuchadas en cada discusión respecto al género. La apuesta ante esta problemática es por un feminismo interseccional, aquel que articule relaciones de sexo, raza y clase social porque la interseccionalidad “consiste en aprehender las relaciones sociales como construcciones simultáneas en distintos órdenes, de clase, género y raza, y en diferentes configuraciones históricas”^48^.

Entre las problematizaciones que se asocian con las intersecciones, el ecofeminismo surge como la posibilidad de relaciones entre el ambientalismo y el feminismo. El último camino que emerge en las reflexiones permanentes que hacen las dos cuentas de Instagram, para enfrentar las violencias machistas es el ecofeminismo, como la fusión entre feminismo y naturaleza.

*…la naturaleza y las mujeres no son tan distintas: son dominadas, violentadas y explotadas por una visión extractivista del mundo, pues ambas son vistas como recursos que se pueden explotar indefinidamente, invisibilizar y no remunerar en su trabajo reproductivo. Es de ahí́ que nace el ecofeminismo*. (@lainsumisa, 11 mayo de 2020, Instagram)

Es necesario aclarar que el ecofeminismo va en contra del ecofascismo, una corriente que defiende que el medio ambiente debe recuperarse sin importar a quién afecte.

El movimiento del ecofeminismo, que surge a principios de los años 80 por la unión de los movimientos pacifistas, ecologistas y feministas, presenta a las mujeres como salvadoras de la tierra, al considerar que se encuentran en mayor armonía con la naturaleza debido a su capacidad de ser madres. Las mujeres son definidas como esencialmente creativas, nutricias y benignas, reivindicando la asociación mujer-naturaleza, históricamente dominada por el binomio hombre-cultura. Este movimiento feminista exalta el principio femenino y sus valores, y propone recuperar la dimensión espiritual de la vida, entendiendo la espiritualidad como el principio femenino que habita e impregna todas las cosas […] La espiritualidad de las mujeres se dispone a *sanar a la madre tierra* y a devolver su magia al mundo, celebrando la dependencia hacia la tierra, a la vez que liberándola de la represión violenta ejercida por los hombres.^49^

El discurso del ecofascismo, por ejemplo, en pandemia, fue expresado por quienes no estaban muriendo o siendo afectados por las consecuencias del covid-19; también por aquellas personas con privilegios: estar cómodos en casa durante la cuarentena, teletrabajar, tener solvencia económica, acceso a la salud, indiferentemente de quienes la pasaron mal, sufrían o murieron. 

Tal como hemos visto, los espacios digitales se afianzan como escenarios relevantes para la lucha por las reivindicaciones de género^50^ a través de los cuales es posible visibilizar problemáticas de alto impacto en la salud física y mental, como los ocasionados por las violencias basadas en género en las sociedades latinoamericanas en el marco del confinamiento por la pandemia de covid-19. 

## CONCLUSIÓN

En las cuentas de Instagram @lainsumisa y @feministasenconstrucción, las problematizaciones predominantes frente a las violencias basadas en género son muy completas y ofrecen apuestas importantes para posibilitar nuevas miradas y caminos hacia la equidad de género, el respeto por la mujer, la educación y reeducación social frente a la prevención de la violencia. 

Estas apuestas del feminismo en redes sociales tienen un alto componente de esperanza en la transformación social y, para ello, no son ajenas al ser, con un especial énfasis en el ser mujer, movilizando desde el discurso hacia las autoresistencias, el amor propio y la sororidad. De igual forma, la mirada está puesta en las dinámicas de la economía del cuidado, la comprensión de la violencia machista, el cuestionamiento del amor romántico, la mirada crítica a la mujer en los medios de comunicación, la televisión, el cine, los cómics y las series.

Dentro de las problematizaciones predominantes que hacen estas cuentas de Instagram, hay lugar para pensar y repensar la visibilidad de la mujer negra y racializada, la interseccionalidad y el ecofeminismo, conceptos que se perciben atravesados por discursos contrahegemónicos y antipatriarcales.

La interacción del público con los contenidos relacionados con la violencia de género, publicados en las cuentas de Instagram @lainsumisa y @feministasenconstrucción durante el primer año de pandemia de covid-19 en Colombia, evidencia cuentas activas, destacadas y seguidas por una cantidad considerable de personas usuarias, también da cuenta de que se han abierto camino en el mundo de las redes sociales de la mano de los conocimientos, deseos y movilizaciones de mujeres artistas, académicas, escritoras, comunicadoras, abogadas, psicólogas, madres, hijas, hermanas, amigas y, en general, mujeres comunes que coincidieron para tejer comunidad pensando en abrirle camino al género y convocar muchas otras mujeres. En este sentido, el ciberactivismo es una oportunidad para el surgimiento de colectivos y redes de apoyo para las mujeres que luchan por la equidad de género y por sus derechos, hacia el cuestionamiento de ideas patriarcales que atentan contra su bienestar.

Por último, con el discurso y postura de ambas cuentas queda claro que entienden que, como en los escenarios sociales físicos, también en los digitales hay posturas, ideas y opiniones diversas y contrapuestas, lo cual es motivo para tejer discusiones, dar lugar a nuevas ideas y entender que existe el punto de vista contrario, en vez de polemizar, asumir el rol de *hater* o pretender tener la razón a toda costa.

A manera de recomendaciones y aportes para el cambio social, se destaca la relevancia de comprender y visibilizar las violencias basadas en género desde una perspectiva de interseccionalidad en la que este tipo de contenidos sean accesibles también, en contextos donde convergen múltiples exclusiones como aquellas en razón de la ubicación geográfica, la situación socioeconómica y los asuntos étnicos y raciales entre otros.

Respecto al ámbito colectivo, también es indispensable posicionar y avanzar en las discusiones acerca de la valorización social de los cuidados como gran pilar del sostenimiento de la vida. Al respecto, los gobiernos y los medios de comunicación tienen mucho por hacer y por reestructurar. Asimismo, urgen políticas contundentes para prevenir y mitigar los impactos sociales, familiares e individuales de las violencias basadas en género como un asunto de salud pública que genera miles de víctimas cada año en sociedades como la colombiana.
